# Impact of Reducing Intake of Red and Processed Meat on Colorectal Cancer Incidence in Germany 2020 to 2050—A Simulation Study

**DOI:** 10.3390/nu15041020

**Published:** 2023-02-17

**Authors:** Tobias Niedermaier, Thomas Gredner, Michael Hoffmeister, Ute Mons, Hermann Brenner

**Affiliations:** 1 Division of Clinical Epidemiology and Aging Research, German Cancer Research Center (DKFZ), 69120 Heidelberg, Germany; 2 Cancer Prevention Unit, German Cancer Research Center (DKFZ), 69120 Heidelberg, Germany; 3 Department of Cardiology, Faculty of Medicine and University Hospital Cologne, University of Cologne, 50931 Cologne, Germany; 4 Division of Preventive Oncology, German Cancer Research Center (DKFZ) and National Center for Tumor Diseases (NCT), 69120 Heidelberg, Germany; 5 German Cancer Consortium (DKTK), German Cancer Research Center (DKFZ), 69120 Heidelberg, Germany

**Keywords:** potential impact fraction, red meat, processed meat, colorectal cancer, policy intervention

## Abstract

Background: According to the International Agency for Research on Cancer (IARC), there is sufficient evidence for the carcinogenicity of processed meat consumption in humans, specifically regarding colorectal cancer (CRC) risk. Evidence for the carcinogenicity of red meat consumption is more limited but points in the same direction. Methods: A macro-simulation approach was used to calculate age- and sex-specific potential impact fractions in a 30-year period (2020–2050). Aims: We estimated numbers and proportions of future CRC cases preventable under different scenarios of reducing the intake of processed and red meat in the German population. Results: Eliminating processed meat intake could reduce the burden of CRC by approximately 205,000 cases in Germany (9.6%) in 2020–2050, 2/3 among males (145,000) and 1/3 among females (60,000). Without red meat intake, approximately 63,000 CRC cases could be avoided (2.9%), 39,000 among males and 24,000 among females. Reductions in the mean consumption of both processed and red meat by one or two servings (each 11 or 22 g) per day would be expected to reduce CRC case numbers by 68,000 (3.1%) and 140,000 (6.5%), respectively. Conclusion: A reduction in red and processed meat intake might substantially reduce the incidence of CRC in Germany. The means of achieving such a reduction might include price and taxation policies, food labeling, and clearer risk communication aiming to reduce individual intake.

## 1. Introduction

Colorectal cancer (CRC) ranks second among the most common cancer-related causes of death, and third among the most common cancers, with approximately 870,000 and 1.1 million new cases among women and men worldwide, respectively, in 2020 [1]. In Germany, CRC is the third most common cancer in terms of incidence, with approximately 60,000 incident cases annually, and the second most common cancer in terms of mortality, with approximately 25,000 deaths per year. Furthermore, it involves substantial treatment costs [2]. The International Agency for Research on Cancer (IARC) concluded in 2018 that there was “sufficient evidence in humans for the carcinogenicity of consumption of processed meat” (Group 1) with respect to CRC [3]. This classification is based on “sufficient evidence” from epidemiological studies. Similarly, red meat was classified as a likely cancer risk factor (Group 2A, probably carcinogenic to humans). This classification is based on “limited evidence” from epidemiological studies (confounding could not be ruled out).

The intake of red or processed meat is high in Germany: According to a nationally representative survey from 2008 to 2011 (German Health Interview and Examination Survey for Adults—DEGS1) [4], the median daily intake is approximately 38 g of red meat and of 46 g of processed meat. The IARC recommends limiting the daily intake of red or processed meat. Every 100 g of red meat intake or 50 g of processed meat intake per day have been associated with an increase in CRC risk by 12% and 16%, respectively [5]. More than a quarter of the adult population consumes more than 108 g of red or processed meat per day (756 g per week) [4], which by far exceeds the recommendation of the German Nutrition Society of 300–600 g per week [6]. A previous study estimated that of all incident CRC cases in 2018, 11.4% could be attributed to processed meat consumption, and 0.7% to red meat consumption [7].

Reducing the amount of red and processed meat consumed is, thus, expected to have a relevant potential to reduce the incidence of CRC in Germany. In this study, we estimated the potential impact of a reduction in or elimination of red or processed meat intake on numbers of CRC cases in the German population from 2020 until 2050.

## 2. Materials and Methods

We applied a macro-simulation approach in which age- and sex-specific potential impact fractions (PIFs) were calculated, based on expected numbers of CRC cases in Germany and preventable CRC cases if changes in dietary habits occurred, i.e., if consumption of red and/or processed meat was reduced or eliminated. Similar to population-attributable fractions (PAFs), PIFs reflect a proportional decline in disease (here: CRC) risk following a partial or complete removal of exposure to one or more cancer risk factors [8]. The following input parameters were used in the simulation model:

### 2.1. Red and Processed Meat Intake

We used self-reported information on the amount and frequency of red and processed meat intake from the DEGS1 survey, a nationally representative survey in which health status and health behavior were assessed among 8152 adults aged 18 to 79 years from Germany in 2008 to 2011. The validated food frequency questionnaire in DEGS1 with 53 items included information on the frequency of different types of red meat (including hamburgers, kebab, all types of pork, beef, and deer) and processed meat (sausages or ham) and the amount of the respective type of meat (number of servings per day). Portion sizes in grams were taken from a Master thesis conducted at the Robert Koch Institute (RKI) [9], from which we derived intake in grams per day.

### 2.2. Expected Numbers of CRC Cases without Intervention

Data on CRC incidence in Germany was taken from the German Centre for Cancer Registry Data (ZfKD), Robert Koch Institute, Berlin [10]. Expected numbers of CRC cases without any intervention were estimated assuming age- and sex-specific incidence rates to remain constant at the levels observed in 2017/2018 and forecasts of the sex- and age-specific population figures (variant 1 assuming moderate changes in fertility, life expectancy, and net immigration) made by the Federal Statistical Office [11].

### 2.3. Risk Estimates

Relative risks (RRs) for the association between red or processed meat intake and risk of CRC were obtained from a published report by the World Cancer Research Fund (WCRF)/International Agency for the Research on Cancer (IARC) [5], according to which CRC risk is increased by 16% for every unit of 50 g of processed meat per day, and by 12% for every unit of 100 g of red meat per day.

Red or processed meat intake, respectively, were assumed to be reduced or eliminated over a 5-year period (2020–2024). CRC risk reductions following a reduction in red or processed meat intake were not assumed to take effect immediately. Instead, the concepts of latency times (LAT) and lag times (LAG) were applied [12]. During the LAT time, cancer risk remained constant after cancer risk factor exposure changed. During the subsequent LAG time, risk gradually changed (here: decreased) until the risk of individuals not exposed to the risk factor was reached (relative risk [RR] = 1). In our main analysis, we assumed a LAT time of 1 year and a LAG time of 9 years. In sensitivity analyses, the LAG time was varied between 5 and 15 years. Those durations are reasonable estimates based on the previously reported time for development from preclinical cancer to manifest CRC of approximately 5 years [13], plus approximately 5 years that are required for the development of CRC from adenomas [14,15,16].

### 2.4. Statistical Analysis

Let RR_rm_ be the relative risk of CRC per 100 g red meat consumed per day, and RR_pm_ be the relative risk per 50 g processed meat consumed per day.

According to the WCRF/AICR estimates [5], RR_pm_ and RR_rm_ were assumed to be 1.16 and 1.12, respectively. Let M_pm_ and M_rm_ be the mean consumption of processed or red meat in each individual, respectively. Assuming a log–linear relationship between consumption of red or processed meat and CRC risk, the RRs for individual consumption levels (RR_pm, i_ and RR_rm, i_) compared to no consumption were computed as follows:

RR_rm, i_ = exp (M_rm, i_ • ln (RR_rm_)/100)

RR_pm, i_ = exp (M_pm, i_ • ln (RR_pm_)/50)

For example, if an individual consumes 50 *g* of red meat, this individual RR_rm, i_ would be exp(50 • ln(1.16)/100) = 1.08. Then, the potential impact fraction (PIF) of the incidence resulting from the modification in the processed or red meat consumption was calculated, using the “RR shift” method [8]:

$$PIF=1-\sum_{i}^{n}{RR}_{i}\left(M_{i}^{*}\right)/\sum_{i}^{n}{RR}_{i}\left(M_{i}\right)$$

where $M_{i}$ is the consumption level of processed or red meat of individual i, ${RR}_{i}$ is the corresponding time-dependent CRC relative risk for that individual (which we assumed to be independent of age in the absence of age-dependent RRs), and $M_{i}^{*}$ is the modified consumption level of individual i in a specific intervention scenario. If, for example, everyone consumed an amount of processed meat corresponding to an RR of 1.16 (=50 g), and that amount was reduced by 50%, the corresponding PIF would be 1 − (1.08/1.16) = 0.07, or 7%.

For our simulation scenarios, sex- and age-specific (5-year age groups) estimates of PIF were multiplied with predicted sex- and age-specific numbers of CRC cases in the absence of any specific intervention, assuming constant incidence rates over time at the level of 2020.

We examined the following interventions: reduction in mean red meat intake by one serving per week (75 g per week or approximately 10.7 g per day), reduction by two servings per week, and elimination of red meat intake. Analogous scenarios were examined for processed meat intake, assuming the same serving sizes. All analyses were performed with the statistical software R [17] version 4.1.3.

## 3. Results

Between 2008 and 2011, the age-standardized mean amount (frequency multiplied with the amount per serving) of red and processed meat intake in grams per day according to the nationally representative DEGS1 survey was as follows: men, 47 g and 61 g, respectively; women, 29 g and 32 g, respectively (Table 1). The intake of red and processed meat was higher among males than among females in all age groups. More than 500 g of red meat per week was consumed by 5% of the females and 13% of the males, and more than 150 g of processed meat was consumed by 52% of women and 80% of men.

Without any intervention, approximately 1.2 million CRC cases are expected to occur among men between 2020 and 2050, and approximately 940,000 cases among women, of which approximately 15% (men) and 9% (women) can statistically be attributed to the intake of processed and red meat (Table 2).

**Figure 1:**
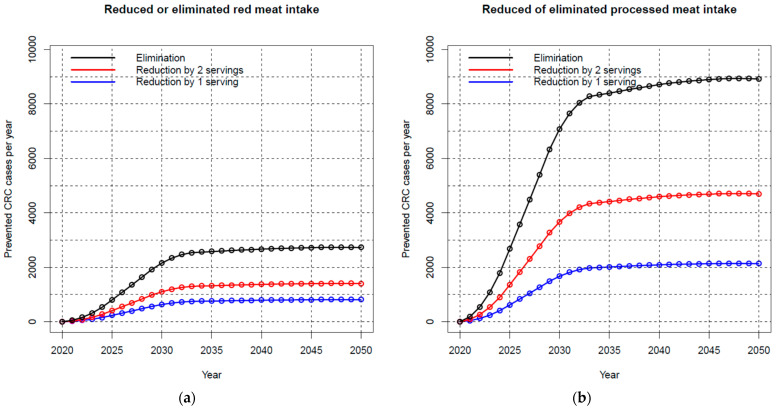
Prevented colorectal cancer cases per year in case of a reduction in consumption of (**a**) red meat and (**b**) processed meat (men and women combined). Abbreviations: CRC, colorectal cancer.

Figure 1a shows the potential impact fractions (i.e., preventable proportions of cases) multiplied with expected case numbers, thus the expected preventable numbers of cases, for CRC in the investigated time frame in the scenario of reduced or eliminated red meat consumption. By assumption, annual preventable case numbers would gradually increase in the initial 10 years due to the assumed latency (1 year) and lag (9 years) time. Thereafter, numbers would remain largely constant and vary only due to projected differences in the expected case numbers resulting from demographic changes. A permanent decrease in red meat intake by only one serving per day could reduce the annual number of CRC cases by almost 500 among men and by more than 300 among women (Figure S1). Two servings less would correspond to almost twice those numbers. Eliminating the risk factor “red meat” could prevent more than 1700 cases among men and 1000 cases among women every year in the long term in Germany.

Figure 1b shows the corresponding numbers for processed meat. Numbers of preventable CRC cases per year would be much higher than for red meat because of the higher RR associated with processed meat intake compared to the same amount of red meat. More than 6000 cases among men and more than 2500 among women could eventually be prevented per year by eliminating processed meat consumption, with correspondingly lower reductions if consumption was reduced by only two or only one serving per day (Figure S2).

**Figure 2:**
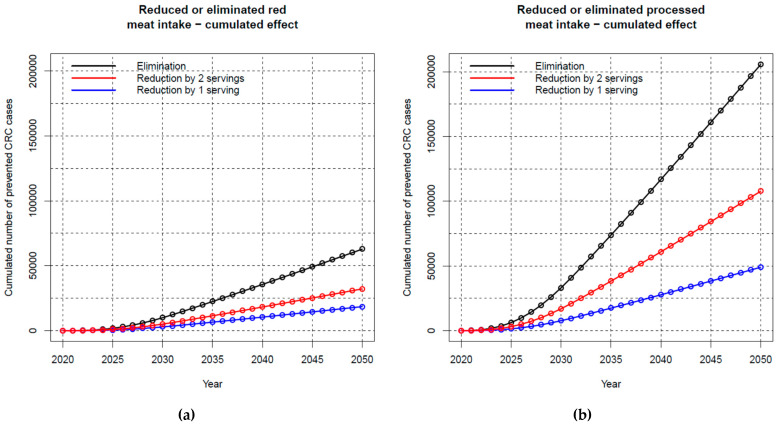
Cumulative prevented colorectal cancer cases over time in case of a reduction in (**a**) red and (**b**) processed meat consumption (men and women combined). Abbreviations: CRC, colorectal cancer.

The cumulative effect of reduced red meat intake on CRC incidence is shown in Figure 2a and summarized in Table 2. Over the entire study period (until 2050), numbers of preventable CRC cases with eliminated red meat intake would sum up to almost 40,000 among men and more than 20,000 among women. Reduction would be larger among men than among women because of the higher CRC incidence and meat consumption among men. Reducing the amount of red meat consumed by one serving per day is expected to lower the number of CRC cases in that time frame by almost 19,000 among men and women combined. Two servings less of red meat intake could reduce the number of CRC cases by more than 19,000 among men alone, and almost 13,000 among women, i.e., by approximately 32,000 cases in total.

Reducing processed meat intake by one serving per day was estimated to decrease the number of incident CRC cases by almost 50,000, again with a higher number among men (almost 29,000) than among women (more than 20,000). Higher numbers of preventable CRC cases could be achieved if intake could be reduced by two servings per day (in total more than 100,000, approximately 61,000 among men and 47,000 among women). In the optimistic scenario of an elimination of processed meat intake, almost 206,000 cases of CRC could potentially be avoided until 2050, thereof approximately two thirds among men (Table 2, Figure 2b).

Sensitivity analyses using shorter or longer lag periods did not change the results materially. Estimated numbers of preventable CRC cases were slightly lower if a longer lag period of 15 years was assumed and slightly higher if a shorter lag period of 5 years was assumed (by approximately 10% each).

## 4. Discussion

In this study, we used a macro-simulation modeling approach to simulate the decrease in future incident CRC cases associated with a reduction in red and processed meat intake over a 30-year horizon. Different scenarios were investigated, from a reduction by one serving per day over two servings to a total elimination of red and processed meat intake. Overall, our simulation suggests that until 2050, up to almost 63,000 CRC cases (2.9% of all CRC cases) could be avoided if red meat intake were eliminated and almost 220,000 cases (9.6% of all CRC cases) if processed meat intake were decreased to zero. In the conservative scenario of a reduction by one serving per day, approximately 19,000 (red meat) and 49,000 (processed meat) CRC cases could be prevented. A reduction by two servings per day would result in those numbers approximately doubling.

Notably, “sufficient evidence for a causal link” between intake of processed meat (and “limited evidence” for a causal link between red meat) and CRC risk does not indicate particularly strong associations. Most likely, substantially more CRC cases could be prevented by increasing screening uptake and physical activity, and by a reduced prevalence of smoking, alcohol consumption, and overweight or obesity. Nevertheless, carcinogenesis is a multifactorial phenomenon, and awareness of certain types of meat being now considered established (processed meat) and suggested (red meat) CRC risk factors, respectively, should be increased.

Suggested mechanisms by which the intake of red and processed meat increase CRC risk include *N-*nitrose compounds [18,19] and lipid peroxidation [19]. Furthermore, heterocyclic aromatic amines (HAAs) are produced when cooking meat at high temperature [3]. Future studies might investigate whether CRC risk associated with red and processed meat intake differs and could potentially be reduced by different manufacturing techniques (e.g., reduction or avoidance of nitrous compounds in processed meat) or consumption (e.g., by cooking with lower temperatures, avoiding open fire, or removing burnt parts of red meat). Until the underlying mechanisms are clarified, the safest way of avoiding the excess CRC risk associated with processed and red meat intake is avoidance or at least reducing intake of those foods. In addition, this would also be in line with the planetary health diet as proposed by the EAT-Lancet Commission, which is mainly plant-based and flexitarian and recommends greatly limiting meat consumption to achieve a healthy and, at the same time, environmentally sustainable diet [20]. Meat production is more energy-intense than that of plant-based foods, and is the most important source of methane, which is a potent greenhouse gas [21].

Few other studies published between 2017 and 2019 [22,23,24,25,26] have investigated the burden of CRC related to red and/or processed meat consumption: de Vries et al. [22] estimated that eliminating red and processed meat intake could reduce CRC incidence in Colombia by approximately 13% (males, red meat), 10% (females, red meat), 14% (males, processed meat), and 13% (females, processed meat). Those numbers are higher compared to our study, apparently because of the higher intake of red and processed meat in Colombia compared to Germany. Similarly, a study from Denmark with methodology comparable to ours [23] and also using a 30-year time period (2016–2045) found that an elimination of red and processed meat (combined) could prevent almost 17,000 CRC cases, or almost 20% of all cases. Again, this percentage was higher than in our study (also when considering red and processed meat combined), apparently because red meat intake in Denmark is almost twice as high as in Germany. In Canada, 0.9% and 0.7% of all cancers in 2015 were attributed to red and processed meat intake, respectively [26], using comparable methods. A study from France [24] suggested that 19 disability-adjusted life years (DALYs) per 100,000 people were associated with red meat consumption for CRC. More DALYs (21/100,000) were expected to be contributed by the association between red meat consumption and cardiovascular disease. Finally, a cost-effectiveness study from the US [25] pointed to methods of how to potentially achieve the desired reduction in red and processed meat consumption. The authors suggested that an excise tax and warning labels would be highly cost-saving (not only cost-effective) and substantially reduce cancer burden.

Our study adds to the growing body of evidence on preventable cancer cases if recommendations regarding physical activity and body weight [7], smoking [27], alcohol consumption [28], and dietary habits [7] were adhered to. However, the aforementioned estimations of PIFs and population-attributable fractions (PAFs) did not consider correlations between risk factors. Attributable numbers of cancer cases (with different hypothetical interventions to decrease them) should, thus, instead be used to rank their relative importance and priority to address them, and not to sum them up across different risk factors. Additionally, from a public health perspective, the focus should be broadened from CRC risk as a relevant health outcome associated with red and processed meat intake to all relevant health outcomes, since red and processed meat intake have also been consistently suggested to increase risk of cardiovascular disease [29,30,31] and even all-cause mortality [29,30,31].

Similar to tobacco control policies, a combination of interventions would most likely be favorable in order to achieve a long-term decrease in red and processed meat intake, targeting both the supply and the demand side: First, risks of red and processed meat intake should be communicated clearly, specifically the difference between certainty of evidence and potential population impact (association strength and prevalence). Even though it is only partly under the control of the responsible authorities, misunderstandings such as those caused by the IARC/WCRF report, [5] that was widely—and falsely—understood as red and processed meat being similarly dangerous with respect to cancer risk as smoking, should be avoided. Nevertheless, the current fourth edition of the European Code Against Cancer [32] includes the avoidance of processed meat and the limitation of red meat intake as part of the recommendation for a healthy diet. Second, as demonstrated by tobacco control policies, price increases are highly effective in reducing demand. Taxation of red and processed meat beyond current levels of value-added tax would most likely also be very effective in reducing red and processed meat consumption [33]. Other types of “nudging” to decrease meat consumption (e.g., proposing “veggie days” in company canteens) are also an option, but entail the risk of exerting unintended opposite effects by causing psychological reactance.

Another approach could be to set the right incentives (“make the healthy choice the easy choice”) [34]. This could include the span of policies similar to those used for tobacco control, e.g., taxation and warning labels, but also other measures such as food labeling are conceivable. For example, mandatory information about production facilities (space and average life-span per animal, use of antibiotics, etc.) on meat products could influence consumer perceptions and would probably reduce meat consumption. On the other hand, measures to improve the conditions under which meat is produced (e.g., more space per animal) would lower consumption by resulting in higher prices. A unit tax (e.g., per 100 g of meat) would likely be preferable over an ad valorem tax, because the latter might shift demand towards lower-priced low-quality meat, unless minimum prices are specified.

Red and processed meat intake are currently not the main drivers of cancer incidence worldwide and also not mentioned in the latest Global Burden of Disease Study 2019 study report [35]. Nevertheless, as the prevalence of other risk factors such as smoking decreases in Germany and many other countries, the relevance of red and processed meat intake is likely to remain substantial despite recent slight decreases in meat intake in Germany.

This study has several important strengths. It is—to our knowledge—the first study to model the potential impact of a reduced intake of red and processed meat on CRC incidence in Germany. We used nationally representative data on red and processed meat consumption. Strengths of associations were taken from the best currently available evidence (WCRF report). Lag and latency periods were considered in order to avoid an overestimation of effects. Sensitivity analyses assuming longer or shorter lag periods did not affect the results materially, suggesting that our findings are robust with respect to key modeling assumptions.

Our study also has limitations. Risks associated with red and processed meat intake are most likely substantially influenced by the way it is processed (temperature, duration, use of open fire, and use of nitrates and heme iron and lipids that are involved in lipid peroxidation), which could not be investigated in detail due to a lack of data. Our calculations, therefore, instead correspond to the estimated impact of a reduction in intake of “average” red and processed meat. Assumptions regarding latency and lag times from exposure to red and processed meat to CRC development had to be made since they are not precisely known. However, the impact of those assumptions was small. Finally, data on meat intake from the DEGS1 survey was self-reported, and was collected from 2008 to 2011, more than 10 years ago. Even though meat consumption has remained fairly stable in the past 10 years in Germany [36], recent slight decreases would imply that estimated numbers of future preventable CRC cases were slightly overestimated. Such a potential, though very small, of overestimation of potentially achievable effects would not affect our results qualitatively or any of its implications.

More studies and data are required to assess potential differences in impact on cancer incidence according to different ages at exposure and a reduction/elimination of exposure. For example, it is unclear if exposure to the risk factor “processed meat consumption” (and potentially red meat consumption) increases CRC risk at all ages similarly (in which case an exposure criterion analogous to smoking pack–years could be established) or if exposure is more relevant at younger or at older ages.

In future studies, it should be investigated in more detail if the risk increase associated with red and processed meat intake is modified by other CRC risk and protective factors such as smoking. Such information could be valuable for the calculation of individual CRC risk, for instance, in risk scores. Furthermore, potential substitution effects should also be carefully assessed. For example, the intake of red and processed meat would most likely not simply be reduced ceteris paribus (all else equal) as we assumed in our study, but go along with an increase in demand for other foods. Ideally, those would be “healthy foods” such as high-fiber vegetables and whole-grain products, and/or lead to a reduction in calorie intake and, thus, contribute to a reduction in overweight and obesity in the population. Increased vegetable and whole grain intake would further reduce CRC risk. In a pessimistic scenario, reduced intake of red and processed meat would be compensated by higher intake of energy-dense foods such as fried vegetables, sweets, etc., that would promote another common CRC risk factor (obesity). “Health-risk-adapted” taxation of foods could guide substitution in the desired direction, not only with respect to CRC risk.

Future studies should assess the means by which a reduction in red and processed meat intake could be achieved most efficiently (i.e., at the lowest costs from a societal perspective) in Germany, be it unit or ad valorem taxes and/or subsidies for more healthy alternatives. One possibility would be applying the regular value-added tax (currently 19%) to red and processed meat rather than the reduced rate (currently 7%) that applies to “essential goods”. In return, more healthy foods that are associated with lower risk of CRC and other diseases such as fruits, vegetables, and whole-grain products could be exempt from taxation.

Given that producing and consuming red and processed meat is and almost certainly will always be legal in Germany, one could question the legitimacy of measures aiming to decrease consumption. However, similar measures have been undertaken for other non-essential and harmful products such as tobacco and alcohol. Considering the high treatment costs of CRC in Germany [37,38], the taxation of products increasing the risk of developing CRC or other cancers would rather reflect an internalization of externalities. To be clear: We do not propose that red and processed meat should be banned, which would be incompatible with a liberal democratic society. However, from a public and planetary health perspective, it seems justified to aim to change food taxation in order to shift demand, to some extent, in the direction of healthier foods and a diet with a smaller environmental footprint.

## 5. Conclusions

In summary, a reduction in red and processed meat intake would most likely have a modest, but non-negligible positive impact on CRC incidence in Germany, with approximately 205,000 (processed meat) and 63,000 (red meat) preventable cases in 2020–2050. The optimal way to achieve such a reduction (e.g., a combination of taxation and subsidies, among other measures) needs to be elucidated by further research. More education about the health risks associated with red and processed meat intake and further preventive efforts are warranted.

## Figures and Tables

**Table 1:** Summary statistics of red and processed meat intake (grams/day) in Germany according to the DEGS1 survey among 8152 adults aged 18 to 79 conducted between 2008 and 2011, overall, by sex, and by 5-year age categories.

	Males	Females	Overall	18–24	25–29	30–34	35–39	40–44	45–49	50–54	55–59	60–64	65–69	70–74	75–79
Red meat intake															
Minimum	0	0	0	0	0	0	0	0	0	0	0	0	0	0	0
1st quartile	11	11	11	11	11	11	13	11	11	11	11	11	11	11	11
Median	26	26	26	26	26	26	26	26	26	26	26	26	26	26	26
Mean	47	29	38	45	39	39	41	38	37	37	33	35	34	29	28
3rd quartile	60	30	60	60	60	60	60	60	60	60	60	60	60	30	30
99th perc.	240	120	189	240	240	240	240	120	120	120	120	120	120	120	120
Processed meat intake															
Minimum	0	0	0	0	0	0	0	0	0	0	0	0	0	0	0
1st quartile	24	11	16	19	16	18	19	17	17	17	17	14	14	15	12
Median	47	23	33	42	37	38	40	36	35	34	32	27	29	27	26
Mean	61	32	46	55	50	55	54	47	47	46	44	39	39	35	36
3rd quartile	78	42	59	74	66	66	73	63	62	60	55	52	54	48	45
99th perc.	242	169	220	239	199	219	244	213	236	199	511	169	172	147	171
Red and processed meat intake															
Minimum	0	0	0	0	0	0	0	0	0	0	0	0	0	0	0
1st quartile	54	28	37	41	35	39	44	40	41	40	37	36	35	35	32
Median	89	48	68	77	66	70	75	73	69	72	65	65	63	56	52
Mean	107	61	84	100	88	95	95	86	84	83	76	74	73	65	64
3rd quartile	135	81	108	133	123	119	122	111	106	110	101	97	97	85	85
99th perc.	415	233	340	478	410	341	378	309	356	323	275	231	278	203	235

Abbreviations: perc., percentile; DEGS1, first wave of the “study on health among adults in Germany”.

**Table 2:** Estimated number and proportion of colorectal cancer cases preventable under different theoretical scenarios of reducing red and processed meat intake over a 30-year period (2020–2050) in the German population, stratified by sex.

	Expected Number of Cancer Cases in the Absence of Changes	Total (#) and Relative (%) Number of Prevented Colorectal Cancer Cases Per Scenario											
Sex Analysis		-1 Serving Red Meat Per Day		-2 Servings Red Meat Per Day		Elimination of Red Meat Intake		-1 Serving Processed Meat Per Day		-2 Servings Processed Meat Per Day		Elimination of Processed Meat Intake	
		#	%	#	%	#	%	#	%	#	%	#	%
Men													
Main analysis ^1^	1,208,329	10,890	0.9	19,292	1.6	39,178	3.2	28,633	2.4	60,721	5.0	145,291	12.0
Lag time: 5 years		11,718	1.0	20,763	1.7	42,183	3.5	30,809	2.5	65,334	5.4	156,403	12.9
Lag time: 15 years		9611	0.8	17,019	1.4	34,541	2.9	25,268	2.1	53,586	4.4	128,182	10.6
Women													
Main analysis ^1^	939,932	7641	0.8	12,925	1.4	23,611	2.5	20,381	2.2	46,975	5.0	60,273	6.4
Lag time: 5 years		8224	0.9	13,927	1.5	25,446	2.7	21,960	2.3	50,607	5.4	64,978	6.9
Lag time: 15 years		6732	0.7	11,387	1.2	20,800	2.2	17,989	1.9	41,401	4.4	53,073	5.6

^1^ Red or processed meat intake, respectively, is assumed to be reduced or eliminated over a 5-year period (2020–2024). Assuming a lag period of 9 years and a latency period of 1 year if not stated otherwise.

## Data Availability

The data supporting the findings of this manuscript are publicly available as described in the section “Materials and Methods”.

## Supplementary Materials

The following supporting information can be downloaded at: https://www.mdpi.com/article/10.3390/nu15041020/s1, Figure S1: Prevented colorectal cancer cases per year in case of a reduction in red meat consump-tion, stratified by sex.; Figure S2: Prevented colorectal cancer cases per year in case of a reduction in processed meat consumption, stratified by sex.
