# Supplementary material for: Impact of Reducing Intake of Red and Processed Meat on Colorectal Cancer Incidence in Germany 2020 to 2050—A Simulation Study
Source: Nutrients. 2023 Feb 17;15(4):1020. doi: 10.3390/nu15041020 (PMC9966277; doi:10.3390/nu15041020)
Supplement: Supplementary file 1 [file nutrients-15-01020-s001.zip › Niedermaier et al. - Supplementary Figure S1.pdf]

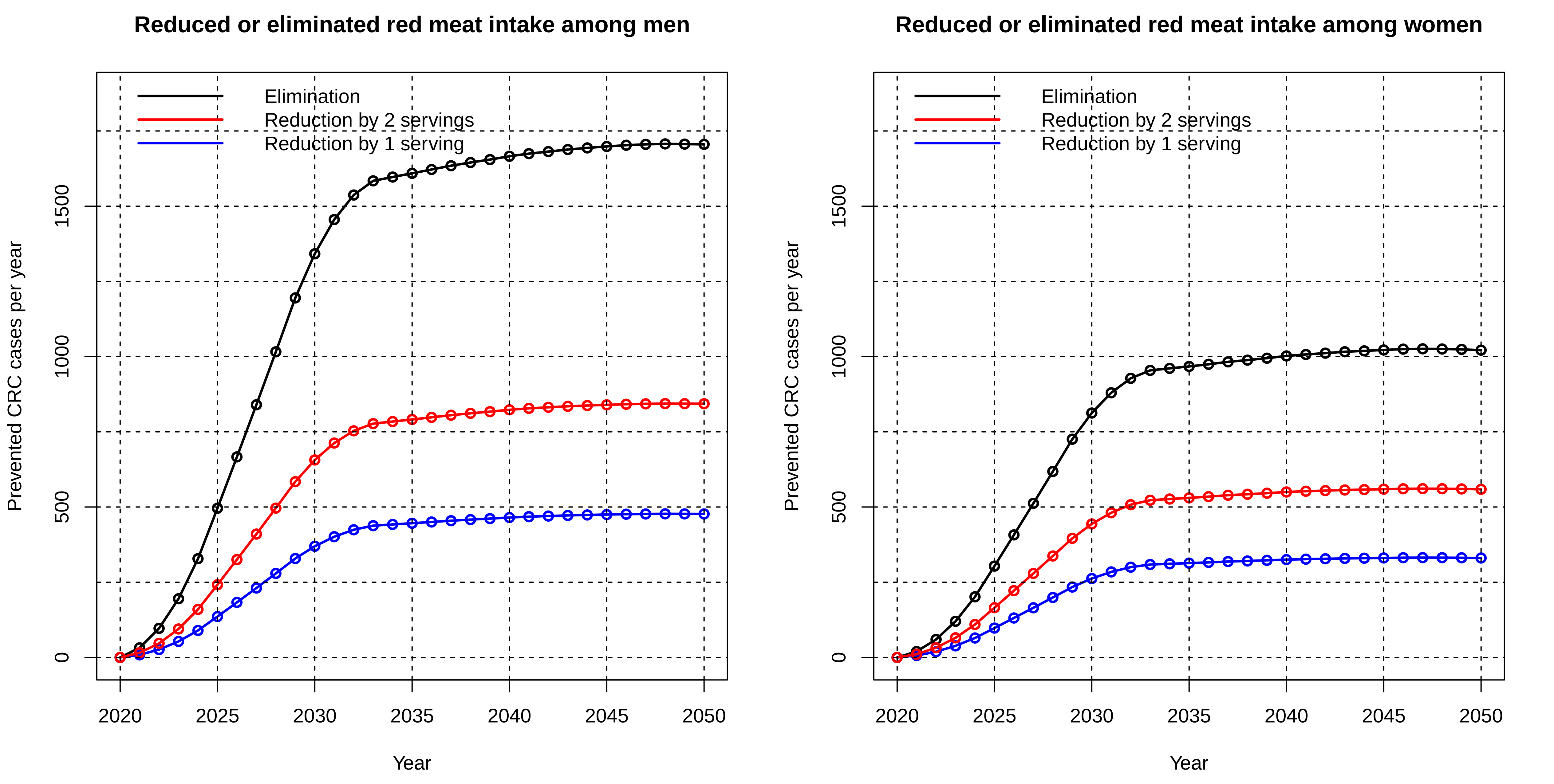

Reduced or eliminated red meat intake among men
Elimination
Reduction by 2 servings
Reduction by 1 serving
Prevented CRC cases per year
0
500
1000
1500
2020
2025
2030
2035
2040
2045
2050
Year
Reduced or eliminated red meat intake among women
Elimination
Reduction by 2 servings
Reduction by 1 serving
Prevented CRC cases per year
0
500
1000
1500
2020
2025
2030
2035
2040
2045
2050
Year
